# Histone Acetylase Inhibitor Curcumin Impairs Mouse Spermiogenesis–An *In Vitro* Study

**DOI:** 10.1371/journal.pone.0048673

**Published:** 2012-11-07

**Authors:** Xiaoyu Xia, Heng Cai, Shixiao Qin, Chen Xu

**Affiliations:** 1 Department of Histology & Embryology, Shanghai Jiao Tong University School of Medicine, Shanghai, China; 2 Shanghai Key Laboratory of Reproductive Medicine, Shanghai, China; Institute of Zoology, Chinese Academy of Sciences, China

## Abstract

In the previous study, we unraveled the unique “erasure strategy” during the mouse spermiogenesis. Chromatin associated proteins sequentially disassociated from the spermatid chromosome, which led to the termination of transcription in elongating spermatids. By this process, a relatively naïve paternal chromatin was generated, which might be essential for the zygotic development. We supposed the regulation of histone acetylation played an important role throughout this “erasure” process. In order to verify this hypothesis, we treated mouse spermatids *in vitro* by histone acetylase (HAT) inhibitor Curcumin. Our results showed an inhibiting effect of Curcumin on the growth of germ cell line in a dose-dependent manner. Accordingly, the apoptosis of primary haploid spermtids was increased by Curcumin treatment. As expected, the acetylated histone level was downregulated. Furthermore, we found the transcription in spermatids ceased in advance, the dynamics of chromatin associated factors was disturbed by Curcumin treatment. The regulation of histone acetylation should be one of the core reprogramming mechanisms during the spermiogenesis. The reproductive toxicity of Curcumin needs to be thoroughly investigated, which is crucial for its further clinical application.

## Introduction

Spermatogenesis is a complex process of differentiation, involving the self-renewal and proliferation of spermatogonia, the meiosis of spermatocytes, and the spermiogenesis happened to the spermatids [1]. All these events in seminiferous tubules were under the influence of spermatogenic niche which is mainly formed by Sertoli cells. At last, morphological and biochemical specialized spermatozoa were formed. The whole process is regulated by both extrinsic stimuli and intrinsic gene expression. Any impairment to this highly organized program, either in spermatogenic cells or in the testicular somatic cells, might result in male infertility or potential birth defects.

During spermiogenesis, haploid round spermatids undergo a series of changes, ending with the production of extremely differentiated spermatozoa. Based on their morphological features, developing spermtids are divided into Step 1–16 in mice [2]. One unique feature of spermiogenesis is the restart of transcription in haploid spermatids. In previous study [3], we confirmed by an *in vitro* run-on assay that transcription continued in Step 1–7 round spermatids, but gradually decreased in Step 8–9, which was finally shut down at Step 10. The transcriptional product of this period could be very important for the later spermatid development, even for the fertilization and early embryogenesis. It should be noticed that transcription was terminated long after meiosis completed so as it was not coupled to cell cycles.

In order to explore the cause of transcription cessation in spermatids, we detected the dynamics of representative transcriptional factors and regulators throughout the spermiogenesis. We found these proteins removed from the chromatin synchronously with the transcription silence. In addition, an extensive range of chromatin associated factors (CAFs), including essential transcription factors and regulators, remodeling factors, epigenetic modifiers, were found mostly departed from the chromatin before Step 9. In conclusion, during the reprogramming of spermiogenesis, there was a finely orchestrated dissociation of types of CAFs, which might contribute directly to the closure of transcription. This process could erase the paternal epigenetic pattern and generate a relative naïve chromatin. A much similar erasure program was also observed in the late oogenesis [4]. Taken together, this reprogramming during gametogenesis would be essential for the installation of the zygotic developmental program after fertilization. At this moment, the regulation of this erasure procedure was mostly unknown.

In another aspect, histone modifications dynamically modulate chromatin structure, conducting the chromatin binding of functional molecules. We wonder if the disassociation of CAFs is causally related to the changes of epigenome in spermatids. Generally, acetylation of histones, especially acetylated histone H3 and H4 (AcH3 and AcH4), are considered as markers of “open” configuration of chromatin. During mouse spermiogenesis, the substantial expression of AcH4 was observed in step 1–8 round spermatids, followed by a global hyperacetylation in Step 9–12 elongating spermatids ([5], Figure S1). A similar hyperacetylation wave of histones was also found in the rat elongating spermatids [6]. This characteristic phenomenon has long been understood as a prelude of histone replacement carried by transition proteins (TPs) and protamine, by which the paternal genome packaged into a highly compact structure. In mouse elongating spermatids, the spatial distribution of acetylated H4 within the nuclei was tightly associated with the chromatin condensation.

It should be noticed that, the time point of CAFs dissociation and transcription termination was just before the beginning of histone hyperacetylation. So the “erasure” in spermiogenesis was not a direct consequence of histone replacement, but related to that histone acetylation. In that case, disturbing the acetylation level might injure the programmed spermiogenesis. This view has been preliminarily proved by histone deacetylase (HDAC) inhibitor TSA treatment [7], [8]. However, we believe the execution of histone acetylase (HAT) inhibitors, underlying an induced hypoacetylation status, should be more harmful to the spermatids. In this study, we treated primary mouse spermatids with HAT inhibitor Curcumin *in vitro*, evaluated its effects on cell viability, transcription activity and CAFs dynamics. Our data revealed that, a given dose of Curcumin could upregulate the spermatids apoptosis, as well as accelerated the erasure program happened to the CAFs and transcription, the mouse spermiogenesis was impaired by Curcumin treatment. Therefore, the potential reproductive toxicity of Curcumin, especially for its new preparations, should be carefully investigated.

## Results

### Curcumin Affected the Growth of C18-4 Cell Line in a Dose-dependent Manner

**Figure 1:**
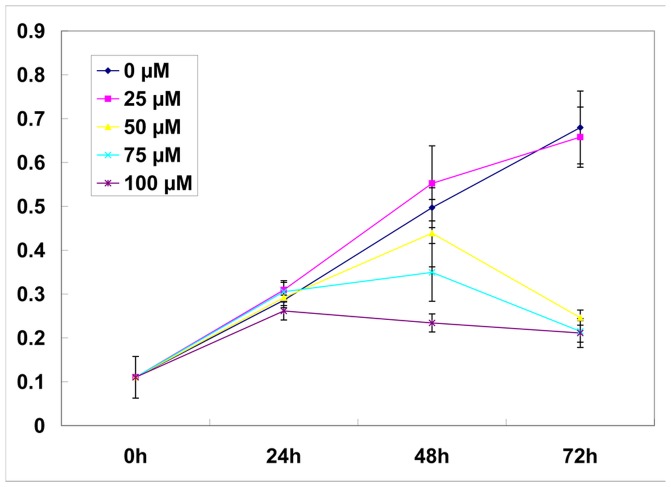
Effects on proliferation of C18-4 cell line by gradient Curcumin treatment. The relative cell number was reflected by MTS assay (Mean ± SD, n = 3). At successive time point, the relative cell number in the 25-µM Curcumin group was similar to that in the control group (0 µM Curcumin) (*p*>0.05). In the 50-µM Curcumin group, cell reduction became apparent at 72 h (*p*<0.05).In the 75-µM and 100-µM groups, the negative effect by Curcumin was manifest since 48 h (*p*<0.05).

Curcumin was proved to be an inhibitor of HAT [9]. To explore the possible impact of Curcumin on spermatogenic cells, we firstly assessed its effect on the proliferation of C18-4 spermatogonia cell line at different doses. The C18-4 cell line, a mouse spermatogonial stem cell line, was established by Dr. Martin Dym’s team at Georgetown University [10]. In our tests, C18-4 cells were cultured in Germ Cell Media containing 0, 25, 50, 75, 100 µM Curcumin respectively. After treatment for 0 h, 24 h, 48 h, 72 h, the proliferation rate of each group was indirectly measured by MTS Assay. As shown in Figure 1, at successive time point, the relative cell number in the 25-µM Curcumin group was similar to that in the control group (0 µM Curcumin) (*p>*0.05). However, in the 50-µM Curcumin group, cell reduction appeared by 48 h and became apparent at 72 h (*p*<0.05); in the 75-µM and 100-µM groups, the negative effect by Curcumin was manifest since 48 h (*p<*0.05). Combining these series of results, we found Curcumin treatment led to a loss of cell viability in a dose-dependent manner. In that context, we applied a moderate treatment of 50 µM Curcumin *in vitro* in the following tests.

### Apoptosis was Induced in Primary Haploid Spermatids by Curcumin Treatment

**Figure 2:**
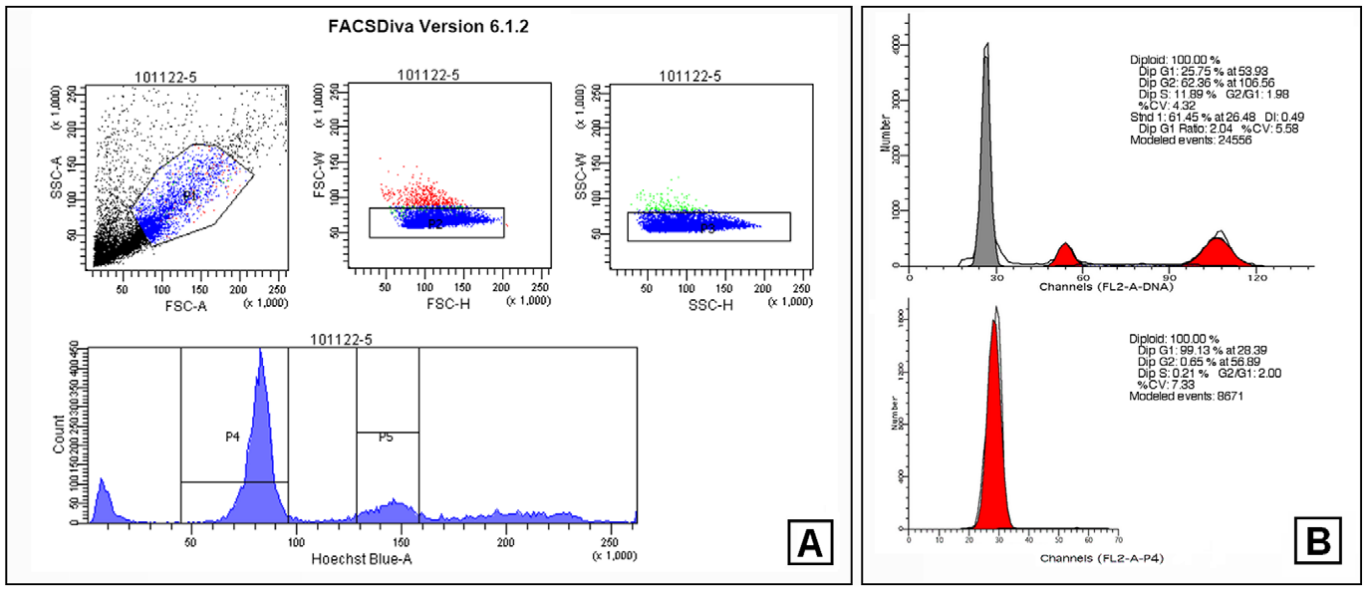
One representative assay of haploid spermatids aggregating by FACS. (A). Hoechst33342 stained primary testicular cells were analyzed in SORP FACSAria II FACScan flow cytometer. **P4** indicated the haploid cell population. (B). The gathered haploid spermatids were confirmed by DNA content analysis (lower) with the total testicular cells used as a control (upper).

**Figure 3:**
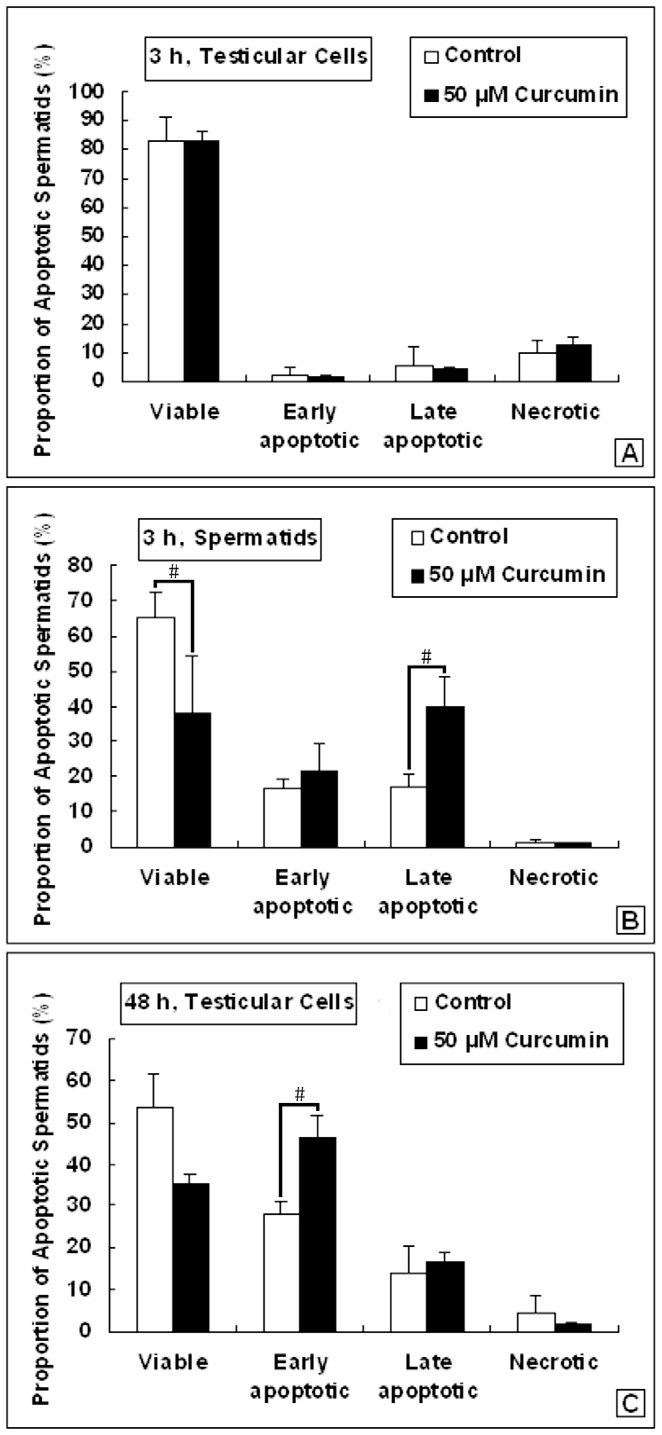
Apoptosis analysis of male germ cells after Curcumin treatment. (A). Apoptosis of testicular cells treated with 50 µM Curcumin for 3 h (Mean ± SD, n = 3). (B). Apoptosis of spermatids treated with 50 µM Curcumin for 3 h (Mean ± SD, n = 3). #*p<*0.05. (C). Apoptosis of testicular cells treated with 50 µM Curcumin for 48 h (Mean ± SD, n = 4). #*p<*0.05.

The primary testicular cells were prepared and treated by 50 µM Curcumin for 3 h or 48 h. The apoptosis analysis was carried out immediately after the treatment. Otherwise, the haploid spermatids were sorted by FACS before the apoptosis analysis. In this study, the purity of haploid spermatids we obtained was stably >95%, certificating the reliability of the following tests (Figure 2). In a 3 h assay for total testicular cells, there was no significant difference between the control and the Curcumin-treated groups (Figure 3. A). However, if we examined merely on the haploid spermatids, there was an obvious increasing of apoptosis in the treated ones (Figure 3. B). Using a 48 h assay, increased apoptosis was observed in the treated testicular cells (Figure 3. C). It seemed that, haploid spermatids were more susceptible to Curcumin treatment than other testicular cell types (Figure 3, Figure S2), implying the regulation of acetylation has much importance in spermatids.

### The Acetylated Histone Level in Spermatids was Downregulated after Curcumin Treatment

**Figure 4:**
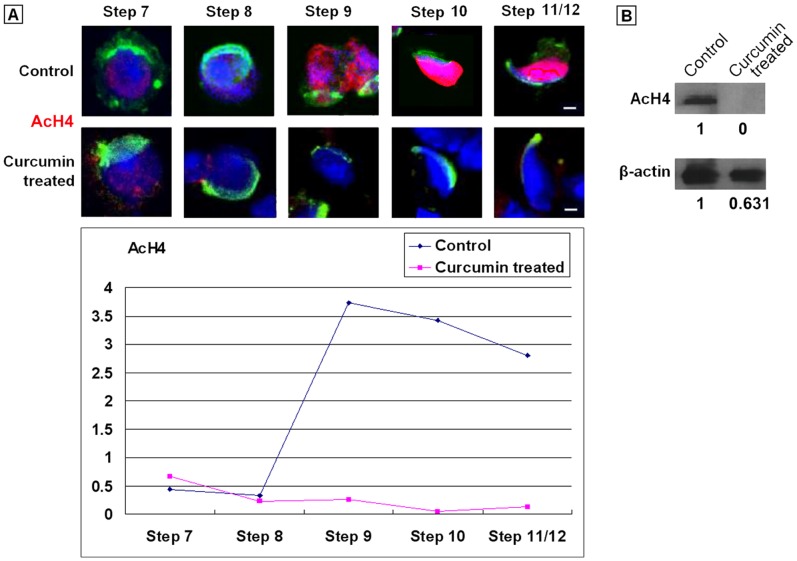
Representative pattern of AcH4 expression in spermatids treated with 50 µM Curcumin for 48 **h.** (A). Immunostaining of AcH4 in spermatids. Step: Developmental steps of spermiogensis. Red: Signals of AcH4. Green: Acrosomes highlighted with lectin PNA. Blue: Nuclei counterstained by Hoechst 33342. Bars = 5 µm. The quantitative analysis of Figure 6 was listed in Table S3. (B). Immunoblot of AcH4 in spermatids.

In normal testis, there was a constitutive expression of AcH4 among Step 1–8 spermatids, followed by a peak of hyperacetylation in elongating spermatids. After 50 µM Curcumin treatment, we detected the expression of AcH4 in the FACS-sorted spermatids. The developmental steps of spermatids were classified according to the published standards, which based on the morphological features, especially the shape of acrosome [2]. We counted at least 30 spermatids at each step. In a 3 h test, no changes were observed for the AcH4 pattern by immunofluorescence staining (data not shown). By contrast, in a 48 h test, the signal of AcH4 retained in Step 1–7 spermatids, but completely disappeared after Step 8, distinct from that in the control group (Figure 4.A, Table S3).

This result was further confirmed by Western blot assay. As we expected, after 50 µM Curcumin treatment for 48 h, the signal of AcH4 was drastically weakened in the experimental group (Figure 4. B). However, there was also a noticeable decrease in the expression level of the internal control protein β-actin. One feasible explanation was that, we loaded protein samples from the same amount of cells onto the each lane of SDS-PAGE gel, but not at the consistent protein concentration. Therefore, a relative long-term Curcumin treatment might negatively affect the global protein levels, involving a rather complex mechanism. As a result, the abnormal acetylation level would lead to a failure of histone replacement and later nuclear condensation. Eventually, the spermatogenesis would be hampered.

### Curcumin Influenced the mRNA Expression of Histone Acetylases and Deacetylases

After Curcumin treatment, we found an abrupt absence of AcH4 signal after Step 8. We assumed that, there was HAT specifically responsible for the hyperacetylation wave in Step 9–12 spermatids, becoming the direct or indirect target of Curcumin treatment. Aim to confirm the inferred HATs, we detected the mRNA levels of several critical enzymes in spermatids by quantitative real-time PCR (qPCR). The products of *Cdyl*, *Cbp* and *Myst4* are HATs, while *Hdac1* and *Hdac4* encode HDACs belonging to different families. Among these genes, *Cdyl* [11], [12] and *Myst4* [13] were considered testis-specific.

**Figure 5:**
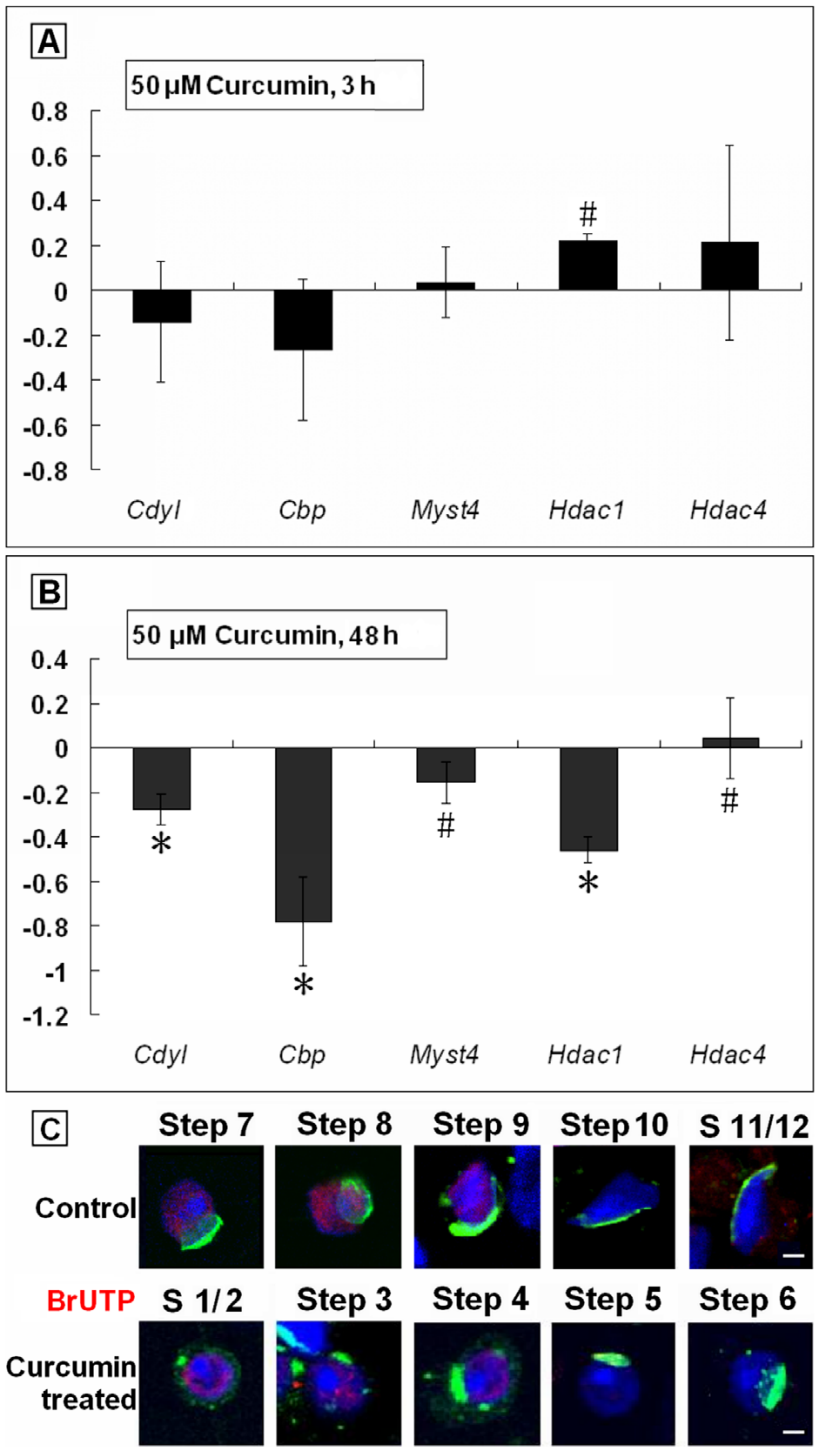
Transcription was affected by 50 µM Curcumin treatment. (A). Quantitative RT-PCR results of mRNA levels of tested genes. Spermatids were incubated with 50 µM Curcumin for 3 h before qPCR executed (Mean ± SD, n = 3). #*p<*0.05. (B). Quantitative RT-PCR results of mRNA levels of tested genes. Spermatids were incubated with 50 µM Curcumin for 48 h before qPCR executed (Mean ± SD, n = 3). #*p<*0.05. *p<0.01. (C). Global transcription status illustrated by *in vitro* run-on assay. Step: Developmental steps of spermiogenesis. Red: Signals of BrUTP incorporation. Green: Acrosomes highlighted with lectin PNA. Blue: Nuclei counterstained by Hoechst 33342. Bars = 5 µm.

Our results showed that, in a 3 h test, the mRNA expression of HAT genes *Cdyl* and *Cbp* seemed to decline in the treated group (*p>*0.05). In contrast to *Cdyl* and *Cbp*, the mRNA level of *Hdac4* tended to go up (*p>*0.05), but actually raised for *Hdac1* (*p<*0.05). For *Myst4*, there was no meaningful changes observed (*p>*0.05) (Figure 5. A). Surprisingly, in a 48 h test, all the detected genes were downregulated (*p<*0.05), except *Hdac4* (Figure 5. B). Our results indicated, these detected genes were truly transcripted in spermatids, and responsed to the Curcumin treatment independently. Thus, the products of *Cdyl*, *Cbp* and *Myst4* might be involved in the normal histone hyperacetylation process.

### Transcription Ceased Earlier in Spermatids Due to Curcumin Treatment

By an *in vitro* run-on assay, we next verified a severe inhibition of transcription in haploid spermatids by Curcumin treatment. In the experimental group, the signal of BrUTP vanished from Step 5 spermtids, much earlier than that in the control (Figure 5. C, [3]), which should be the direct reason for the decreased expression of given genes. Concerning this disorder emerged before the downregulation of histone acetylation, we believed, to some degree, that the Curcumin could affect the transcription apparatus without the mediator functions of acetylated histones.

### The Dynamics of Types of Chromatin Associated Factors were Disturbed by Curcumin Treatment

**Figure 6:**
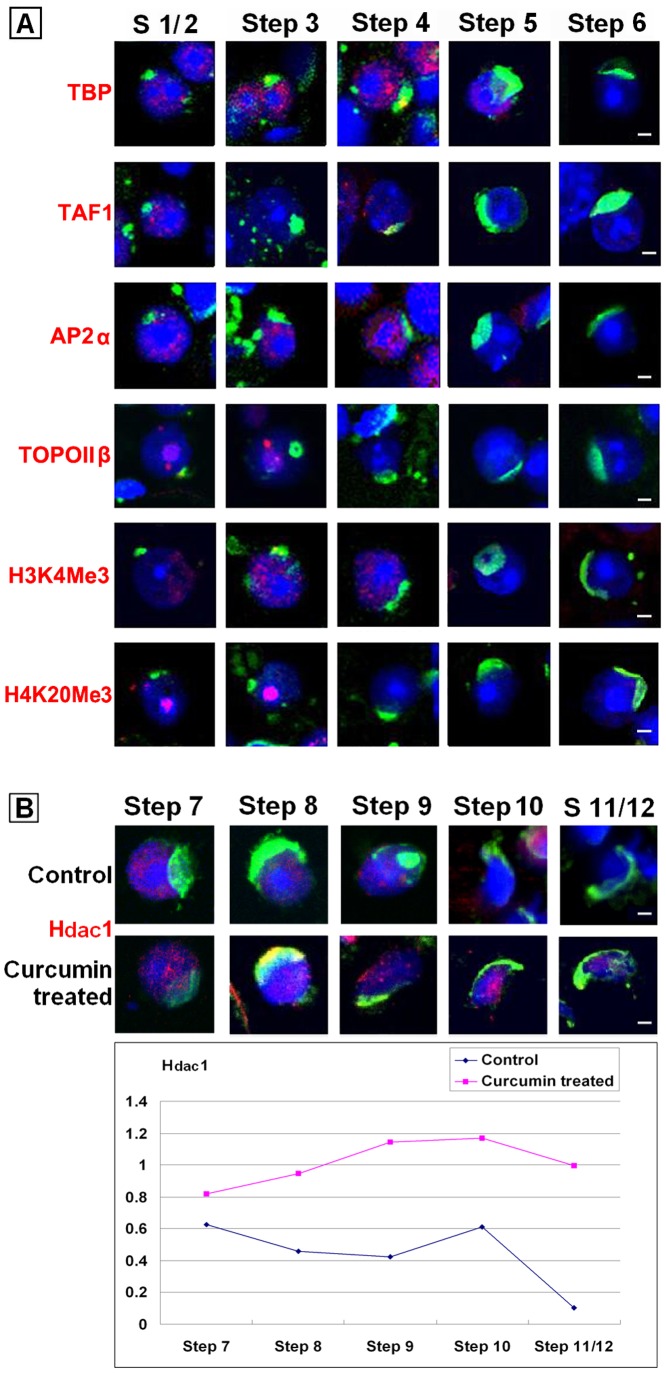
Representative patterns of chromatin associated factors expression in spermatids treated with 50 µM Curcumin for 48 **h.** (A). Immunostaining of TBP, TAF1, AP2α, TOPOIIβ, H3K4Me3 and H4K20Me3 in Curcumin-treated spermatids. Red: Signals of given target. Green: Acrosomes highlighted with lectin PNA. Blue: Nuclei counterstained by Hoechst 33342. Bars = 5 µm. (B). Immunostaining of Hdac1 in Curcumin-treated spermatids. Red: Signals of given target. Green: Acrosomes highlighted with lectin PNA. Blue: Nuclei counterstained by Hoechst 33342. Bars = 5 µm. The quantitative analysis of Figure 6 was listed in Table S3.

In that sense, we were going to examine the dynamics of vital CAFs after Curcumin treatment. By immunofluorescence tests, we found the basal transcription factors TBP and TAF1 disappeared earlier from the spermatids nuclei (Figure 6.A, Table 1), which was coordinated with the fact of disrupted transcription. Then we identified the same pattern to the transcription regulator AP2α, remodeling factor TOPOIIβ, and to epigenetic markers H3K4Me3 and H4K20Me3, all of which were cleared before transcription silence (Figure 6.A, Table 1). These phenomena implied the crosstalk between different epigenetic modifications.

**Table 1:** Summary on the dynamics changes of CAFs during spermiogenesis after 50 µM Curcumin for 48 h.

**Target**	**Function/Implication**	**S** a **1/2**	**3**	**4**	**5**	**6**	**7**	**8**	**9**	**10**	**11/12**	**13/14**	**Test** c	**Control** c
**BrUTP**	Transcription in process	+^b^	+/−b	+/−	−b	−	−	−	−	−	−	−	S1–4	S1–9
**GC sequence**	Euchromatin	+	+	+	+	+	+	+	−/+ b	−/+	+/−	+	S1–14	S1–14
**TP2**	Heterochromatin	−	−	−	−	−	−	−	+	+/−	+/−	+/−	S9–14	S9–14
**AcH4**	Active gene region	+	+	+	+	+	+	+/−	−	−	−	−	S1–8	S1–16
**H3K4Me3**	Active gene region	+	+	+	−	−	−	−	−	−	−	−	S1–3	S1–10
**H4K20Me3**	Repressed gene region	+	+	−	−	−	−	−	−	−	−	−	S1–2	S1–12
**Hdac1**	Histone deacetylase	+	+	+	+/−	−/+	−/+	+/−	+/−	+/−	−/+	−/+	S1–14	S1–9
**TOPOIIβ**	Topoisomerase	+	+	−	−	−	−	−	−	−	−	−	S1–2	S1–8
**TBP**	Basal transcription factor	+	+	+	+/−	+/−	−	−	−	−	−	−	S1–6	S1–8
**TAF1**	Basal transcription factor	+	−	−	−	−	−	−	−	−	−	−	S1	S1–10
**AP2α**	Transcription regulator	+	+	+	−	−	−	−	−	−	−	−	S1–3	S1–9

a Steps of spermiogenesis.

b +: signals positive; −: signals negative; +/−: signals positive in most cells (*>*50%) of given step; −/+: signals negative in most cells (>50%) of given step.

c Test: summary on expression profile of given factors in Curcumin-treated group; Control: summary on expression profile of given factors in normal spermiogenesis.

At the same time, signals of GC sequence and TP2 remained in elongated spermatids, indiscriminating to those in normal spermiogenesis (figure not shown, see Table 1). Considering the anti-GC antibody recognized the euchromatin compartment, it was shown that, our results were not derived from a premature chromatin condensation, or the limitation of our protocols. For those spermatids in which histones had been replaced by transition proteins, the binding of TP2 seemed unaffected. In fact, acetylation of TP2 would lead to a significant reduction in its property of DNA condensation [14].

But there was an outstanding exception, the acetylation modifier Hdac1. In normal spermiogenesis, it seemed undetectable after Step 9 [3], [12]. However, in the Curcumin- treated group, the signal of Hdac1 persisted in Step 1–14 spermatids (Figure 6.B). Although the mRNA expression of *Hdac1* declined after 48 h Curcumin treatment (Figure 5.B), but for the existing Hdac1 protein in spermatids, a HAT-repressed situation seemed enhance their binding to the chromatin. The dynamic of Hdac1 was independent to other detected CAFs. This result strongly suggested the interaction between HATs and HDACs in the spermiogenesis. In a word, the dynamics of kinds of CAFs were obviously disturbed by given Curcumin treatment.

## Discussion

### A Hypothetic Model of Reprogramming in Spermatids Regulated by Histone Acetylation

To explore the mechanism of male fertility, one of the biggest obstacles has been the lack of ideal models of the spermiogenesis, *in vitro* and *in vivo*. In this research, we applied a fluorescence-activated cell sorting based on Hoechst33342 staining to purify the haploid spermatids. Most recently, haploid embryonic stem cell lines were established attributing to the similar ploidy sorting technique [15], [16]. Hoechst33342 is a living-cell permeant and relatively non-toxic [17]. Verapamil has been used as an inhibitor of drug efflux pump proteins to block the efflux of Hoechst33342 [18]. Compared to the regular velocity sedimentation method [19], our approach lead to higher purity with less damage (Figure 2). We obtained a mixture of haploid Step 1–16 spermatids and cultured them for 48 hours. During a classic mouse spermatogenic wave, the round spermatid phase last for 10 days, followed by a 5-days elongating period. Our protocol provides a possibility to analyze the reprogramming in spermiogenesis. However, lost of the nursing from Sertoli cells, the performance of spermatids could be disordered.

Curcumin has been reported as HAT inhibitor [9]. We found Curcumin influenced the proliferation of spermatogonia C18-4 cells in a dose-dependent manner. When C18-4 was incubated with 25 µM Curcumin, an inclination of growth promotion was observed. That was consistent with the previous reports, in which the low concentrations of Curcumin could diminish the ROS generation [20]. However, in our study, when C18-4 was incubated with Curcumin at no less than 50 µM, a growth repression effect became prominent (Figure 1). When we treated primary haploid spermatids with 50 µM Curcumin *in vitro*, the apoptotic level was upregulated even within 3 hours (Figure 3). Our data suggested that, spermatids were vulnerable to the pro-apoptotic effect of Curcumin than other testicular cell types.

In previous research, we disclosed an “erasure” model of mouse spermiogenesis [3]. We postulated that, paternal-zygotic reprogramming begins with a genome-wide clearance of chromatin associated factors (CAFs), to erase the existing program in the spermatids. In present study, we observed a premature CAF disassemble in the Curcumin-treated round spermatids (Figure 6, Table 1), including the basal transcription factors TBP and TAF1, transcription regulator AP2α, remodeling factor TOPOIIβ, and the epigenetic markers H3K4Me3 and H4K20Me3. As a consequence, transcription terminated in advance in the treated spermatids. These findings suggested that, in normal spermiogenesis, the erasure procedure might also be triggered by the hypoacetylation condition. Similar experiments using different dosages of Curcumin would produce more precise details.

We also noticed a sudden disappear of AcH4 signal in Curcumin-treated elongating spermatids. It inferred that, there was peculiar HAT responsible for the histone hyperacetylation in Step 9–12 spermatids, which could be repressed by Curcumin treatment. Then we examined the expression of several HATs: Cbp was proved as substrate of Curcumin [9], Cdyl [11], [12] and Myst4 [13] were reported particularly in elongating spermatids. We revealed a decreased mRNA level of these genes after Curcumin treatment, shown that at least a part of their products were newly synthesized in round spermatids. We had tried to determine their protein levels and dynamics in spermatids, either by Western blot or immunochemistry. Unfortunately, no reliable data was obtained maybe due to their step-specific little content. For the already translated HAT proteins in late round spermatids, their activities might be considerably inhibited by Curcumin treatment. Very importantly, there seemed to be a negative interplay between HATs and Hdac1/HDACs: the newly synthesized HAT, such as Cdyl, might be one direct regulator of HDAC degradation, so that the latter would provide a necessary HDAC-free environment for histone hyperacetylation [11], [12]. Anyhow, the HAT in charge of histone hyperacetylation still requires inspection.

**Figure 7:**
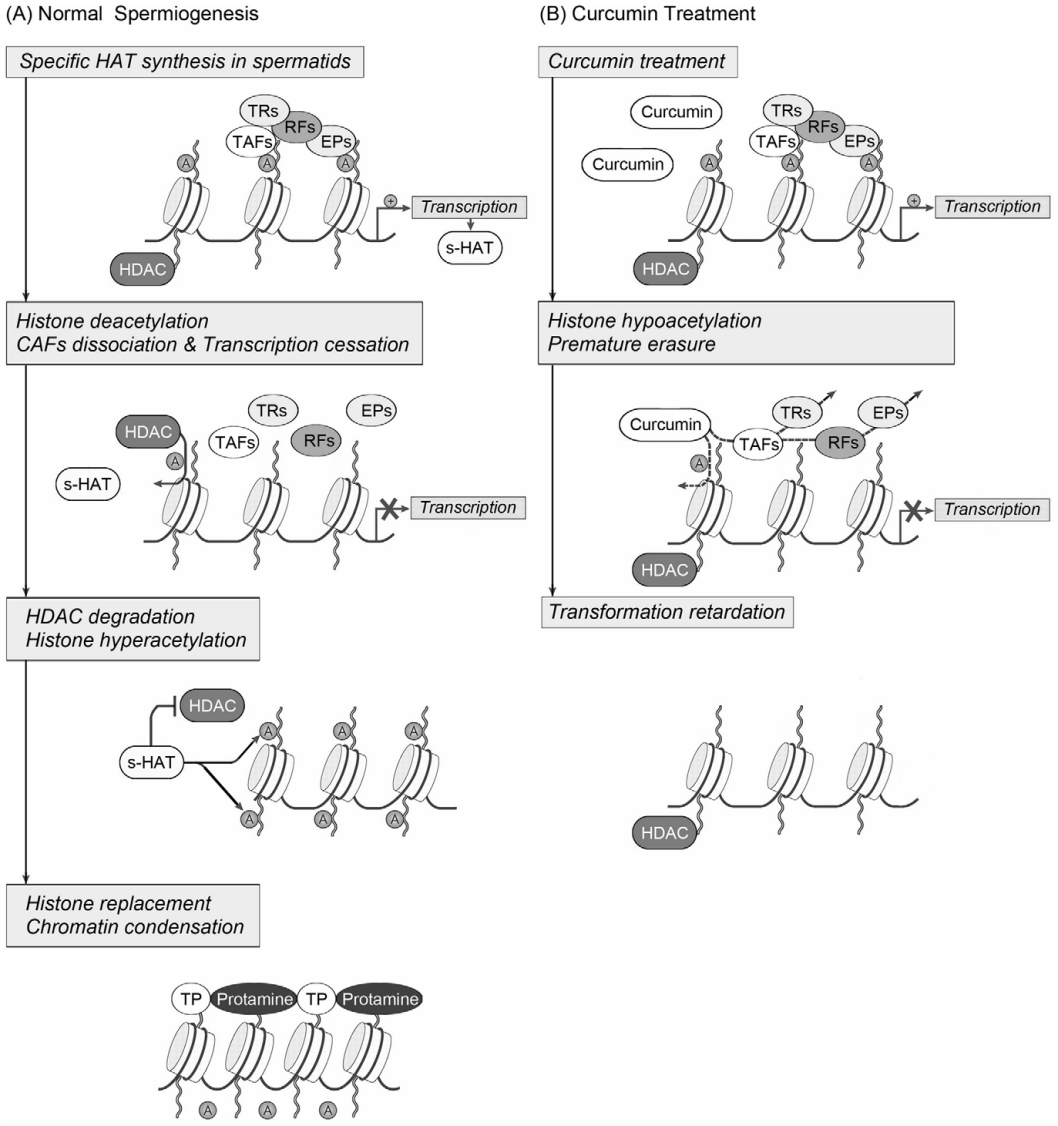
A working hypothesis for the molecular mechanisms of reprogramming in mouse spermiogenesis. (A) During normal spermiogenesis, transcription restarted in spermatids specific HATs were generated. Later, HDACs catalyzed the deacetylation of histones, leading to the CAFs dissociation and transcription cessation. In the meanwhile, the gradually accumulated specific HATs guided the degradation of HDACs, in that case they could induce the histone hyperacetylation in elongating spermatids.The AcH signal then recruited remodeling factors to execute the histone substitution and nuclear condensation. (B) When spermatids were treated with Curcumin, hypoacetylation was induced, resulting in a premature erasure procedure of CAFs and transcription. The spermatid specific HATs were missing, whereas the HDACs retained. At last, the transformation of spermatids was impaired. A: acetylated histones; s-HAT: spermatid specific histone acetylase; HDAC: histone deacetylase; TAFs: transcription associated factors; TRs: transcription regulators; RFs: remodeling factors; EPs: epigenetic modifiers; TPs: transition proteins. (This figure was modified from Tsankova et al. [40]).

Taken together, we put forward a working hypothesis of reprogramming in spermiogenesis as follows (see Figure 7 for illustration): After meiosis, transcription restarted in round spermatids, during this period, some essential and special HATs were generated. Next, HDACs catalyzed the deacetylation of histones, which in turn gave rise to the extensive dissociation of CAFs. Then the transcription finally stopped. The mechanism involved in this erasure process might be universal in gametogenesis [4], [21]. In the meanwhile, the gradually accumulated HATs guided the degradation of HDACs, in that case they could induce the histone hyperacetylation in elongating spermatids. Soon the AcH signal recruited remodeling factors as BRDT [22] to execute the histone substitution and nuclear condensation. This elongating program was spermatogenic-specific. During the reprogramming in spermiogenesis, the regulation of histone acetylation might be central of the controlling network, the balance between HATs and HDACs played an important role. These hypotheses need further investigations.

### Potential Male Reproductive Toxicity of Curcumin

Curcumin is extracted from the plant *Curcuma longa*, has been used as food addictive for thousands of years in Asia. Most recently, it is proved to have a wide range of pharmacological activities, including antioxidant, anti-inflammatory, antiviral, antibacterial, antifungal, and anticancer, as well as a potential that against diverse malignant diseases, diabetes, allergies, arthritis, Alzheimer’s disease, and other chronic illnesses. Curcumin has been listed as third generation cancer chemopreventive agent by the Institution of Cancer Chemoprevention, NCI, NIH of United States [23]. The effects of Curcumin are mediated through a very complex network, the regulation of various transcription factors, growth factors, inflammatory cytokines, protein kinases, and other enzymes [24], whereas we especially concern about its property of HAT inhibitor [9].

Our current knowledge about Curcumin is mainly from researches based on disease models [23]. For example, Curcumin displayed a protective function on testicular tissues under various pathological conditions [25]–[28]. However, its molecular effect on normal tissues or cells has not been sufficiently analyzed. It has been reported that, Curcumin could inhibit human sperm motility, also the capacitation and acrosome reaction [29], [30]. In this study, we proved Curcumin with an impairment effect to mouse spermatogenic cells *in vitro*, since its negative functions on cell viability, CAFs dynamics, transcription activity and acetylated histone regulation.

Furthermore, the optimum utility of Curcumin had long been limited by its low bioavailability caused by poor solubility in aqueous solvents. Until recently, this issue has been improved by the Curcumin-loaded-nanoparticle approach, implying the promising prospect of clinical application [31]. However, at the same time, the problem about the reproductive toxicity of nano-Curcumin is accordingly put forward. There have been batch of evidences on nanoparticles penetrating the blood-testis barrier (BTB) successfully [32]–[35]. So what will happen to the BTB and spermatogenesis by Curcumin nanoparticle treatment? Aim to answer the above questions, we prepared Curcumin-loaded poly (lactide-co-glycolide) nanoparticles (Cur-PLGA for short), and primarily demonstrated that, compared to unformulated Curcumin, Cur-PLAG could accelerate the apoptosis of Sertoli cell line TM4, damage the tight junctions between TM4 cells, thus might be harmful to the BTB *in vivo* (unpublished data). We presume testicular functions more sensitive to the Nano-Curcumin than its conventional forms. To sum up, an *in vivo* application of Curcumin might result in defect of spermatogenesis. The male reproductive toxicology of Curcumin preparations, particularly the nanoparticles, needs to be evaluated prudently. That is also meaningful to the development of male contraceptive drugs in the future.

## Materials and Methods

### Animals

The male ICR mice used in this study were purchased from the Centre for Experimental Animals, Chinese Academy of Sciences, Shanghai, China. The animal procedures were approved by Shanghai Jiao Tong University, School of Medicine, and were conducted in accordance with the National Research Council Guide for Care and Use of Laboratory Animals [SYXK (Shanghai 2007–0025)].

### Cell Culture and Proliferation Assay

Curcumin (Sigma, C7727) was diluted to 0.5 M in DMSO, stored in −20°C until use. The C18-4 cell line was a kind gift from Dr. Martin Dym (Georgetown University Medical Center, USA) which was first established by Dr. Dym’s team in 2005. This cell strain was derived from immortalized mouse type A spermatogonia using the SV40 large T-antigen gene (*LTAg*) under the control of an ecdysone-inducible promoter (more details see Ref [10]). The C18-4 cells were plated onto 96-well plate, cultured in Germ Cell Media [DMEM/F12 containing 15% FBS, 2 mM Glutamine, 50 U/ml Penicillin and 50 µg/ml Streptomycin] at 34°C with 5% CO_2_. Curcumin was added into the media as the final concentration of 0, 25, 50, 75, 100 µM with triplicate wells for each group. After Curcumin treatment for 0 h, 24 h, 48 h, 72 h, the Cell Proliferation MTS Assay was performed according to the manufacture’s protocol (GENMED, GMS10043, China). Briefly, cells were treated with 50 µl working solution per well under dim light. After incubated for 1 h at 37°C with 5% CO_2_, the absorbance of each well was measured at 490 nm using a microplate reader. This experiment was repeated for 3 times.

### Isolation of Testicular Cells and Curcumin Treatment

Testes were detunicated, the seminiferous tubules were collected and minced with sterile scissors in PBS. The tissues were digested in 0.5 mg/ml type IV collagenase and 0.25% trypsin-EDTA in DMEM at 37°C for 15 min sequentially. The released cells were screened through a 200-mesh, culture in Germ Cell Media supplemented with 50 µM Curcumin at 34°C with 5% CO_2_. Control group was cultured without Curcumin treatment. After 3 h or 48 h, cells were collected and washed by centrifugation in PBS. The prepared cells then were used in BrUTP incorporation and immunochemistry detection. For apoptosis assay, Western blot and quantitative real-time PCR (qPCR), haploid spermatids were selected and aggregated by FACS as described below.

### Haploid Spermatids Aggregation by FACS

All the procedures in this part were carried out under sterile conditions. After Curcumin treatment for 3 h or 48 h, testicular cells were transferred into the fresh Germ Cell Media containing 2 µg/ml Hoechst 33342 (Sigma) and 5 µM Verapamil (Sigma), incubated at 34°C for 90 min. The cells were kept on ice before analyzed in a FACScan flow cytometer (SORP FACSAria II). The spermatogenic cell populations were distinguished on the basis of their DNA content [17], [36]. Haploid spermatids (1C) were separated and gathered for the following apoptosis assay, Western blot and qPCR. In our study, the purity of haploid spermatids we harvested was stably >95%, which certificating the reliability of the following tests (see Figure 2). We suggest any drug treatment before the flow cytometry, as the latter might cause injury to the primary cultured cells and influence the final results.

### Apoptosis Assay

The apoptosis analysis was carried out according to the manufacture’s suggestion (Invitrogen, V13241). Concisely, cells were resuspended in 100 µl 1X annexin-binding buffer with 5 µl of Alexa Fluor® 488 annexin V and 1 µl of 100 µg/ml PI working solution, then incubated in dark at room temperature for 15 min. Analysis was performed by flow cytometry (FACS Calibur). This experiment was repeated for 3 times.

For morphological evidence of the apoptosis, the Hoechst 33342/Propidium iodide double staining was carried out. Hoechst 33342 is a blue-fluorescence dye that stains the condensed chromatin in apoptotic cells more brightly than that in normal cells. Propidium iodide (PI), a red-fluorescence dye, is only permeant to dead cells. After the double staining of Hoechst 33342 and PI, the cell population should be separated depending on the compacted state of the chromatin due to the apoptotic status (Figure S2).

### Western Blot Assay

After FACS sorting, the haploid cells were resuspended in 5% SDS solution, incubated at room temperature for 1 h with rotation. After centrifugation at 12,000 rpm for 10 min at 4°C, the supernatant was mixed with sample buffer (Beyotime) and boiled for 5 min. Proteins from approximately 5× 10^5^ haploid cells were loaded onto each lane of 15% SDS-PAGE gel. After electrophoresis, the proteins were electroblotted onto PVDF membrane by Wet Electrophoretic Transfer System (Bio-Rad). The membrane was then blocked with 1× NET [150 mM NaCl, 5 mM EDTA (pH 8.0), 50 mM Tris-HCl (pH 7.5), and 0.05% Triton X-100] for 1 h and incubated with NET-diluted anti-AcH4 antibody, then the horseradish peroxidase conjugated anti-rabbit IgG. β-actin was used as an internal control (see Table S1 for antibody information). The signals were detected with ECL-plus reagents (Millipore). The images were analyzed by Image-Pro Plus 5.02 software.

### RNA Extraction and Quantitative Real-time PCR

Briefly, the sorted haploid spermatids were immediately resuspended in TRIzol reagent (Invitrogen). Total RNA were extracted, reverse-transcription was executed using PrimeScript RT-PCR kit (TakaRa). Primers used in this research were listed in Table S2. The quantitative real-time PCR was performed using the SYBR Premix Ex Taq (TaKaRa). *Gapdh* was used as an internal control. This experiment was repeated for 3 times.

### *In vitro* run-on Assay

The transcription activity in testicular cells was evaluated as described previously [3]. The Curcumin treated cells were washed in physiological buffer (PB) [100 mM potassium acetate, 30 mM KCl, 1 mM MgCl_2_, 10 mM Na_2_HPO_4_, 1 mM ATP supplemented with 1 mM dithiothreitol (DTT), 0.2 mM phenylmethylsulfonyl fluoride (PMSF), and 50 units/ml of RNase inhibitor (Promega)], then transferred to transport buffer (TB) [100 mM potassium acetate, 1 mM MnCl_2_, 50 mM (NH_4_)_2_SO_4_, 30 mM KCl, 10 mM Na_2_HPO_4_ containing 2 mM ATP, 0.4 mM each of guanosine triphosphate (GTP), cytidine triphosphate (CTP), bromouridine triphosphate (BrUTP), and 1 mM MgCl_2_], incubated at 34°C for 30 min. After washed 3 times with PBS, the cells were fixed with 4% PFA for 1 h, smeared on the slides and air-dried. To detect BrUTP incorporation, the slides were incubated with 2 µg/ml anti-BrUTP antibody (Roche) overnight, followed with 0.5 mg/ml CY3-conjugated anti-mouse IgG antibody plus 10 µg/ml Alexa Fluor® 488 conjugated-peanut agglutinin (PNA)(Molecular Probe) [37], [38] for 1 h. After counterstained with Hoechst 33342, the results were examined under a fluorescence microscope (Nikon E600). The developmental steps of spermatids were classified according to published standards [2]. We counted more than 30 spermatids at each step. If the signals were positive in all the counted cells, then recorded as “+” in the Table 1. “−” represent the signals negative in all the counted cells. “+/−” indicates signals positive in most cells (>50%) of a given step, while “−/+” with the opposite meaning.

### Immunofluorescence

Before immunochemistry, testicular cells were prefixed with 0.05% PFA in PBS and decondensed with 10 mM DTT and 0.5 mg/ml heparin for 30 min [39]. The information of primary antibodies was listed in Table S1. The cell suspension from experimental or control group was smeared onto the slides and air-dried, then incubated overnight at 4°C with primary antibodies or corresponding normal serum for negative control. On the second day, cells were washed, incubated with 0.5 mg/ml CY3-conjugated anti-rabbit/goat/mouse IgG antibody plus 10 µg/ml Alexa Fluor® 488 conjugated-PNA for 1 h. After counterstained with Hoechst 33342, the detected signals were counted and summarized as described above. The images in Figure 4.A and Figure 6 were analyzed by Image-Pro Plus 5.02 software. The relative expression intensity of each target protein was calculated as IOD: (Red, Signal)/IOD: (Blue, Nuclear).

### Statistical Analysis

A Student’s t testing was used to analyze the results of cell proliferation assay, apoptosis assay and qPCR. *P* values less than 0.05 (*p<*0.05) were considered statistically significant.

## Supporting Information

Figure S1 **AcH4 expression in normal testicular sections.** Stage: Developmental stages of spermatogenesis. Red: Signals of AcH4. Green: Acrosomes highlighted with lectin PNA. Blue: Nuclei counterstained by Hoechst 33342. PS, pachytene spermatocytes; LS, leptotene spermatocytes; ZS, zygotene spermatocytes; A, intermediate spermatogonia; M, meiotic cells; RS, round spermatids; ES, elongating spermatids. Bars = 10 µm.
(JPG)

Figure S2 **Morphological evidence of apoptosis after Curcumin treatment for 48 h.** Red: Propidium iodide. Blue: Hoechst 33342. Live cells show only a low level of blue fluorescence; apoptotic cells show a higher level of blue fluorescence; dead cells show low-blue and high-red fluorescence. Bars = 100 µm.
(TIF)

Table S1 **Antibodies used in this study.**
(DOC)

Table S2 **Primers used in this study.**
(DOC)

Table S3 **Quantitive analysis of the target protein expression in** **Figure 4** **.A and** **Figure 6** **.**
(XLS)
