# Supplementary figures and images for: Histone Acetylase Inhibitor Curcumin Impairs Mouse Spermiogenesis–An In Vitro Study
Source: PLoS One. 2012 Nov 7;7(11):e48673. doi: 10.1371/journal.pone.0048673 (PMC3492465; doi:10.1371/journal.pone.0048673)

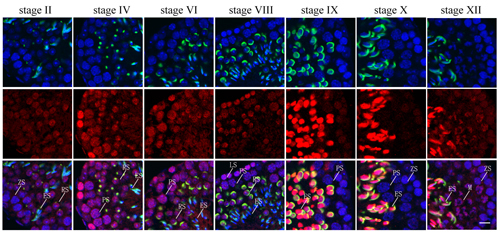

Supplement: Figure S1 — AcH4 expression in normal testicular sections. Stage: Developmental stages of spermatogenesis. Red: Signals of AcH4. Green: Acrosomes highlighted with lectin PNA. Blue: Nuclei counterstained by Hoechst 33342. PS, pachytene spermatocytes; LS, leptotene spermatocytes; ZS, zygotene spermatocytes; A, intermediate spermatogonia; M, meiotic cells; RS, round spermatids; ES, elongating spermatids. Bars = 10 µm. (JPG) [file pone.0048673.s001.jpg]

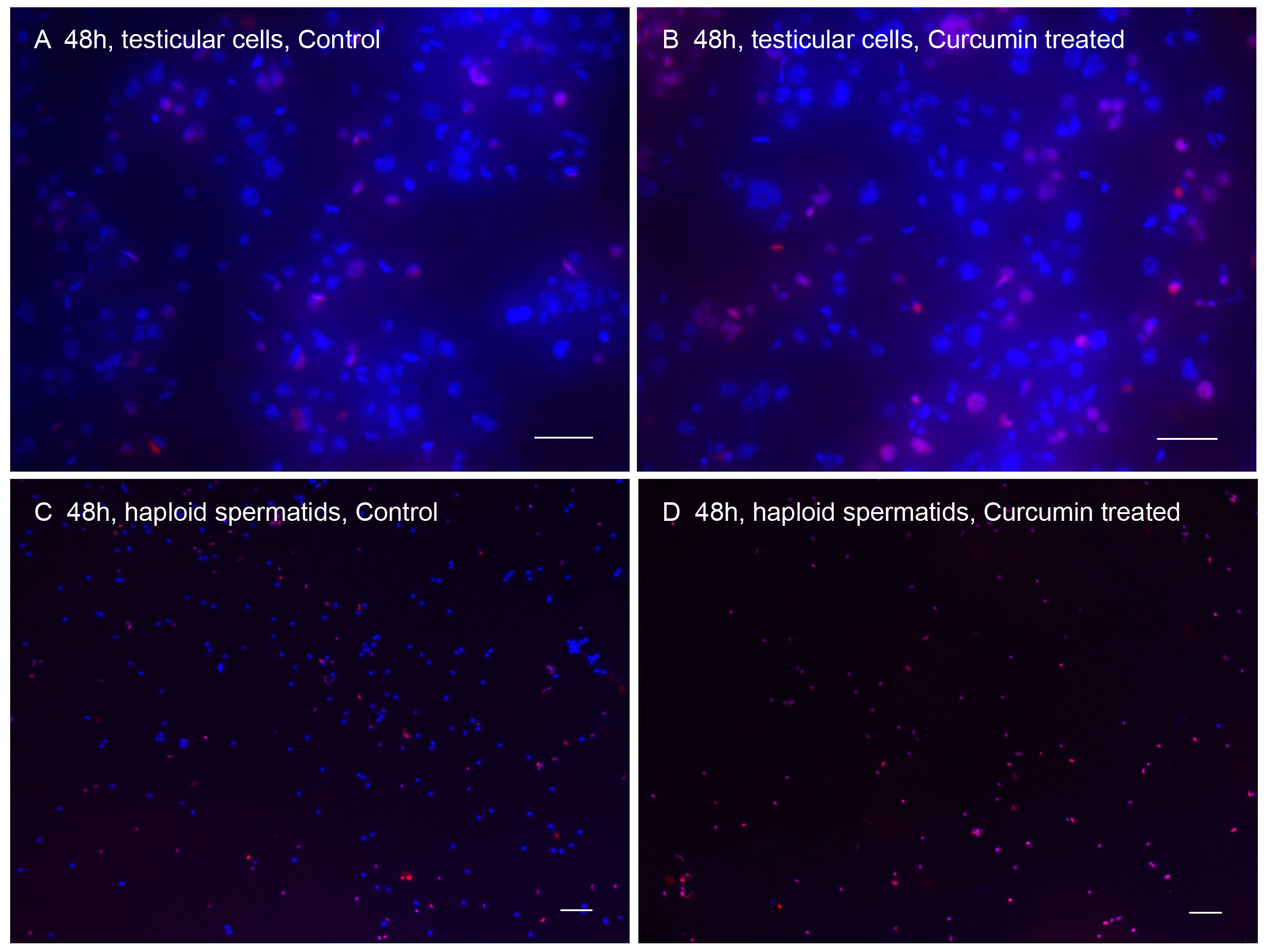

Supplement: Figure S2 — Morphological evidence of apoptosis after Curcumin treatment for 48 h. Red: Propidium iodide. Blue: Hoechst 33342. Live cells show only a low level of blue fluorescence; apoptotic cells show a higher level of blue fluorescence; dead cells show low-blue and high-red fluorescence. Bars = 100 µm. (TIF) [file pone.0048673.s002.tif]
