# Supplementary material for: Histone Acetylase Inhibitor Curcumin Impairs Mouse Spermiogenesis–An In Vitro Study
Source: PLoS One. 2012 Nov 7;7(11):e48673. doi: 10.1371/journal.pone.0048673 (PMC3492465; doi:10.1371/journal.pone.0048673)
Supplement: Table S1 — Antibodies used in this study. (DOC) [file pone.0048673.s003.doc]

**Table S1. Antibodies used in this study.**

| **Target** | **Source** | **Cat. No.** | **Dilution** |
| --- | --- | --- | --- |
| **GC sequence** | Argene | 11-201 | 1:50 |
| **TP2** | Santa Cruz Biotechnology, Inc. | sc-21106 | 1:50 |
| **AcH4** | Upstate | 06-598 | 1:50/1:500**^a^** |
| **H3K4Me3** | Abcam | ab8580 | 1:50 |
| **H4K20Me3** | Abcam | ab9053 | 1:50 |
| **HDAC1** | Santa Cruz Biotechnology, Inc. | sc-8410 | 1:50 |
| **TOPOIIβ** | Santa Cruz Biotechnology, Inc. | sc-13059 | 1:50 |
| **TBP** | Santa Cruz Biotechnology, Inc. | sc-204 | 1:50 |
| **TAF1** | Santa Cruz Biotechnology, Inc. | sc-17134 | 1:50 |
| **AP2α** | Santa Cruz Biotechnology, Inc. | sc-184 | 1:50 |
| **β-actin** | Abcam | ab16039 | −/1:500 **^a^** |

(a): Dilution in immunofluorescence staining/ Western blot assay, respectively.
