# Supplementary material for: Histone Acetylase Inhibitor Curcumin Impairs Mouse Spermiogenesis–An In Vitro Study
Source: PLoS One. 2012 Nov 7;7(11):e48673. doi: 10.1371/journal.pone.0048673 (PMC3492465; doi:10.1371/journal.pone.0048673)
Supplement: Table S2 — Primers used in this study. (DOC) [file pone.0048673.s004.doc]

**Table S2. Primers used in this study**.

| **Genes** | **Sense** | **Antisense** |
| --- | --- | --- |
| ***Cdyl*** | TGACAAAGCAGGTGGTGACCAGAG | GAAGTGTGCAGAGCTGGGCTTC |
| ***Cbp*** | GAACAGCATGGCCTCAGTTCCG | CTCATTGCCCCACTGGATGATGG |
| ***Myst4*** | CAGCAACAAAGGGCAGCAAGCG | TCCCAGCCCATGTGAAGCAACAG |
| ***Hdac1*** | TGCCAAGTGCCTGCTTAGGAGC | TTGCCCAAGATGGGTGGGAAGGT |
| ***Hdac4*** | TTTGTTCCTGCATGTGCTGGTGG | ACCGTGCTGGGCATGTGGTT |
| ***Gadph*** | TCCCACTCTTCCACCTTCGATGC | TGGGATAGGGCCTCTCTTGCTC |
